# Phytochemistry, Bioactivities of Metabolites, and Traditional Uses of *Fagopyrum tataricum*

**DOI:** 10.3390/molecules27207101

**Published:** 2022-10-20

**Authors:** Ivan Kreft, Mateja Germ, Aleksandra Golob, Blanka Vombergar, Alena Vollmannová, Samo Kreft, Zlata Luthar

**Affiliations:** 1 Nutrition Institute, Tržaška 40, SI-1000 Ljubljana, Slovenia; 2 Biotechnical Faculty, University of Ljubljana, SI-1000 Ljubljana, Slovenia; 3 The Education Centre Piramida Maribor, SI-2000 Maribor, Slovenia; 4 Faculty of Biotechnology and Food Sciences, Slovak University of Agriculture in Nitra, Tr. A. Hlinku 2, 949 76 Nitra, Slovakia; 5 Faculty of Pharmacy, University of Ljubljana, SI-1000 Ljubljana, Slovenia

**Keywords:** buckwheat, rutin, quercetin, flavonoids, metabolites, nutrition

## Abstract

In Tartary buckwheat (*Fagopyrum tataricum*), the edible parts are mainly grain and sprouts. Tartary buckwheat contains protecting substances, which make it possible for plants to survive on high altitudes and under strong natural ultraviolet radiation. The diversity and high content of phenolic substances are important for Tartary buckwheat to grow and reproduce under unfriendly environmental effects, diseases, and grazing. These substances are mainly flavonoids (rutin, quercetin, quercitrin, vitexin, catechin, epicatechin and epicatechin gallate), phenolic acids, fagopyrins, and emodin. Synthesis of protecting substances depends on genetic layout and on the environmental conditions, mainly UV radiation and temperature. Flavonoids and their glycosides are among Tartary buckwheat plants bioactive metabolites. Flavonoids are compounds of special interest due to their antioxidant properties and potential in preventing tiredness, diabetes mellitus, oxidative stress, and neurodegenerative disorders such as Parkinson’s disease. During the processing and production of food items, Tartary buckwheat metabolites are subjected to molecular transformations. The main Tartary buckwheat traditional food products are bread, groats, and sprouts.

## 1. Introduction

Tartary buckwheat (*Fagopyrum tataricum* (L.) Gaertn.) originates in the area of the Himalayas. It is cultivated in this mountain region and still exists as well as a wild plant [1,2,3]. Cultivated plants are reproduced in the fields, and local farmers use the crop in their meals. Tartary buckwheat is related to its sister species, common buckwheat (*Fagopyrum esculentum* Moench). Still, it differs in the grain’s much higher concentration of flavonoid rutin and resistance to UV-B radiation [4,5,6]. Tartary buckwheat is the only known field crop with a high concentration of the flavonoid rutin in the grain [7].

Ultraviolet radiation can damage the gentle tissue of plants. There could be a shield from UV radiation by synthesis of protecting substances. These substances are mainly polyphenols, with aromatic rings of six carbon atoms, double ties, and groups bound to carbon atoms, often with attached OH or sugars. In the case of Tartary buckwheat, the most important protecting substances are rutin, quercetin, and fagopyrin [5,8,9,10,11]. Genes and enzymes that make possible the gradual build-up of phenolic substances in Tartary buckwheat have been studied [12].

Tartary buckwheat is cultivated on the Himalayan mountains and elsewhere in the world, mainly in China, Korea, Nepal, Bhutan, and Europe (Ukraine, Russia, Sweden, Luxemburg, Slovenia, Italy, Serbia, Bosnia, and Herzegovina) [13]. In Bosnia and Herzegovina, Tartary buckwheat is mainly cultivated as a mixed crop with common buckwheat [14].

**Figure 1:**
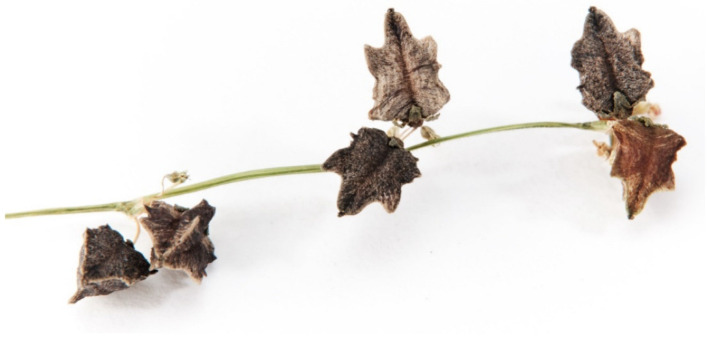
Seeds of Tartary buckwheat.

**Figure 2:**
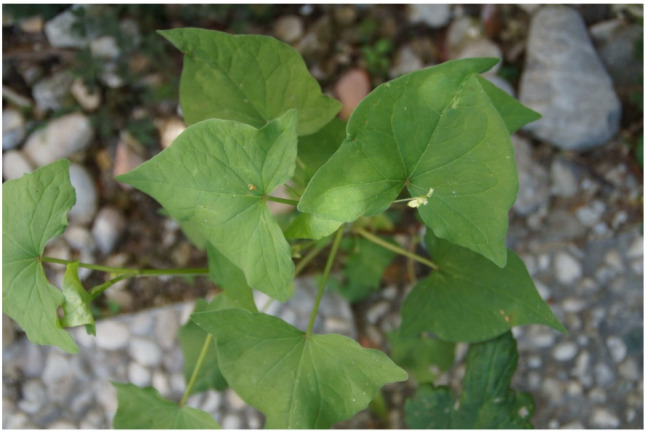
Tartary buckwheat can grow and make seeds even on a ruderal stony area.

Due to its robust husk and high content of protective phenolic substances, dormant Tartary buckwheat seeds may remain alive in the soil for several years and, under favorable conditions, plants can emerge and grow (Figure 1 and Figure 2). Tartary buckwheat, due to its content of protecting substances, survives on high altitudes, under strong ultraviolet radiation, and on stony areas (Figure 2). The diversity and high content of phenolic substances in Tartary buckwheat protect the plants from unfriendly environmental effects, diseases, and grazing. These substances are also of importance in nutrition to preserve human health.

In Tartary buckwheat and in its milling fractions, there are, besides the main phenolic compounds, many other secondary metabolites as volatile aromatic compounds, which may appear as well in Tartary buckwheat food products. They are in low concentrations, so it is not expected that they would have any major bioactive impact of some importance, at least such was not reported according to the knowledge of the present authors [15,16].

The content of total phenols in buckwheat is estimated by the Folin–Ciocalteu method [17]. For the isolation and identification of metabolites, Tartary buckwheat samples are subjected to extraction at room temperature by methanol for 30 min, using an ultrasound bath. Extracted metabolites are fractionated by HPLC using a reverse phase C-18 column. A UV/ VIS detector is included in the system. The components of extract are detected by absorbance at 360 nm. It is suitable to use for mobile phase methanol in the combination methanol-water-acetic acid [5]. Another method of isolation and extraction is shaking for 40 min methanol/water (67:33) at room temperature. HPLC is performed using a Spectra System P4000, Hibar–LiChrospher 100, with a reversed phase RP-18 column. In this case, the solvent for HPLC is acetonitrile, methanol (1:2), and phosphoric acid [18]. Methanol extracts can also be produced by extraction in 80% aqueous methanol. The identification of metabolites is obtained by the reverse phase C18 column. In such a case, the mobile phase consists acetonitrile and 0.1% phosphoric acid in water [19,20]. Among other suitable methods for isolating rutin and other flavonoids is extraction with 60% ethanol and 5% ammonia in water [21]. Further analyses are performed by HPLC or capillary electrophoresis with uncoated capillaries [21].

For the identification of metabolites in diverse Tartary buckwheat tissues, it is possible to develop the potential application of micro-proton induced X-ray emission (micro-PIXE), synchrotron-based micro-X-ray fluorescence (micro-XRF), and inductively coupled plasma-mass spectrometry (ICP-MS) hyphenated with pulsed laser ablation [22]. The results reveal a connection between a plant structure’s morphology and its phytochemical layout with specific bioactivities and functions [22]. With the application of UHPLC-ESI- MS/MS, it is possible to analyze in buckwheat a broad list of metabolites, such as three different phenolic acids, four flavanols, four flavones, seven flavonols, and two flavanones [23,24]). The multimodal bioimaging of Tartary buckwheat for revealing the allocation of metabolites in different parts of Tartary buckwheat plants is under development [22,25,26,27]. Ultra-performance liquid chromatography-electrospray ionization-tandem mass spectrometry multiple reaction monitoring (UPLC-MS/MS MRM) seems to be suitable for determining fagopyrins in Tartary buckwheat extracts as suggested [28]. A novel sensitive electrochemical sensor for rutin determination in Tartary buckwheat was developed recently and will be of much help in the simple and quick determination of rutin in Tartary buckwheat [29]. Other specific methods potentially applied in the determination of bioactive metabolites in buckwheat are reviewed by Huda et al. [30].

Potential bioactivities of Tartary buckwheat and its metabolites are studied: (1) By computer simulation methods. Molecular docking is performed in silico using software and a potential possibility has been found that the buckwheat substance emodin could have a binding affinity to the active sites of the RNA binding domain of the nucleocapsid protein of the COVID-19 virus [31]. According to computer modeling, it was found that hypericin can interact with HIV-1 protease, additional research of the antiviral effects of fagopyrin or other Tartary buckwheat substances similar to hypericin should be performed [32]. (2) In vitro methods are used for studies of the antigenotoxic effect of Tartary buckwheat by induced DNA damage in the human hepatoma cell line, evaluated by the use of the comet assay, and the effects of flavonoids on in vitro Tartary buckwheat starch digestibility [33,34]. (3) Tartary buckwheat reduced the level of low-density lipoprotein cholesterol, total cholesterol, triacylglycerols, glutamic-pyruvic transaminase, glutamic oxaloacetic transaminase, creatinine, urea, uric acid, and malonaldehyde, but increased the level of total protein and the activity of glutathione peroxidase in experimental rats, mice, and piglets [35,36,37]. (4) By epidemiological and clinical studies, the impact of Tartary buckwheat on the prevention of tiredness and cardio-vascular diseases was confirmed [38,39,40,41]. According to the epidemiological study performed in Liangshan, Sichuan, China, Tartary buckwheat nutrition correlated with a lower concentration of low-density-lipoprotein cholesterol, lower total cholesterol, and a higher ratio of serum high-density-lipoprotein in cholesterol in people regularly consuming Tartary buckwheat [41].

## 2. Flavonoids

**Figure 3:**
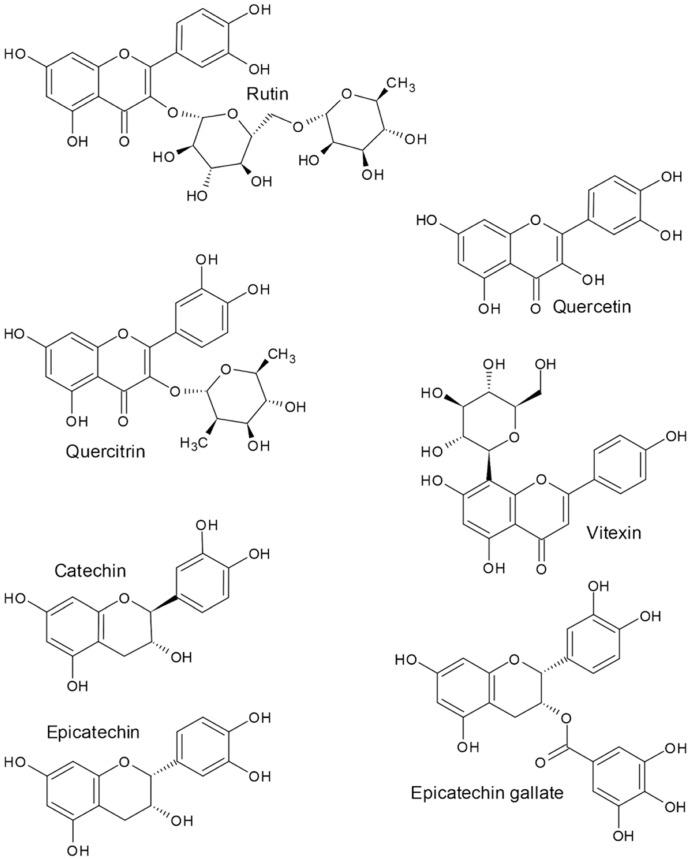
Major buckwheat flavonoids.

Among the important protecting substances of buckwheat are rutin and other flavonoids such as quercetin, quercitrin, vitexin, catechin, epicatechin, and epicatechin gallate (Figure 3). Genes, involved in rutin biosynthesis and regulation were identified in buckwheat [42]. The capability of Tartary buckwheat to tolerate high levels of UV-B radiation and other abiotic stress factors is due to several gene families involved in signal transfer and gene regulation [42]. Researchers identified 769 gene families in phylogenetic trees, which showed the ancestors of Tartary buckwheat [42,43]. The Liangshan Prefecture of Sichuan Province in China is the area famous for cultivating Tartary buckwheat, with the highest content of flavonoids, due to the very high light intensity in the mountain areas. The research in Liangshan reveal that the activity of enzymes involved in the synthesis of flavonoids is positively correlated with the content of Tartary buckwheat flavonoids [44]. High temperatures are affecting the increase of polyphenol and flavonoid content, mainly in the inflorescences, and so it is boosting antioxidant production in Tartary buckwheat plants [45].

However, Zhang et al. [42] defined differences in the Tartary buckwheat that appeared in comparison to its relatives. These differences are obviously responsible for the adaptation of Tartary buckwheat to the environment rich in UV-B radiation and other adverse environmental factors (Figure 2). Genes included in the rutin biosynthetic pathway and the myeloblastosis (MYB) transcription factors were described by Zhou et al. and Zhang et al. [12,42].

Flavonoids and their glycosides are one of the major groups of plant bioactive metabolites. Several bioactive compounds have been detected in various plant parts of buckwheat (roots stem, leaves, flowers, seeds, sprouted seeds, seedlings, seed husks, and processed food of buckwheat) by using different detection methods [46,47,48]. These compounds comprise flavonoids, phenolic acids, and their derivatives, fagopyrins, tannins, triterpenoids, steroids, stilbenes, and so on. Their content depends on various factors including the plant growth stage, organ, cultivated varieties or buckwheat species, growing season, and area [47] (Table 1).

Flavonoids are phenolic compounds, with a 15-carbon skeleton consisting of two benzene rings connected to a heterocyclic pyran or pyrone ring. Rutin (Figure 3) is a flavonol glycoside and quercetin (Figure 3) is its aglycone. In plant and food appearance, quercetin is mainly a result of enzymatic degradation of rutin, due to rutinosidase activity [56,57,58,59,60,61,62]. Flavonoids are compounds of special interest due to their antioxidant properties and potential in preventing tiredness, diabetes mellitus, oxidative stress, and neurodegenerative disorders such as Parkinson’s disease [26,39,49]. Quercetin orally received is able to enter and accumulate in the brain as it can cross the blood–brain barrier [63,64].

One of the activities of rutin in buckwheat plants is the protection of plant tissues and organs from solar UV radiation [65,66]. In addition, quercetin derivatives were detected among the main bioactive substances in buckwheat root exudates, protecting buckwheat plants from weeds [67,68]. The exposure of milled or crushed buckwheat grain to water results in the rutinosidase enzymatic breakdown of rutin to quercetin [69,70,71].

Tartary buckwheat grain contains 0.8 to 2.9% of rutin [8,72]. In the green parts of Tartary buckwheat, there is 0.1% of rutin in young leaves and up to 3.4% in developed leaves (Table 2) [8]. In Tartary buckwheat sprouts, there is approximately 0.3% of rutin in young sprouts and up to 2.5% in developed sprouts [8].

Suzuki et al. [58] reported results from the experiment with rats on the possibilities of toxic effects of rutin-rich dough from Tartary buckwheat by acute and subacute toxicity studies (10,000 and 5000 mg/kg flour per animal body weight, respectively). The concentration of rutin in Tartary buckwheat grain material was 1570 mg/100 g. No toxic symptoms were found. The reports of Suzuki et al. and Vogrinčič et al. [85,86] confirmed that Tartary buckwheat grain flour was not genotoxic.

## 3. Phenolic Acids

**Figure 4:**
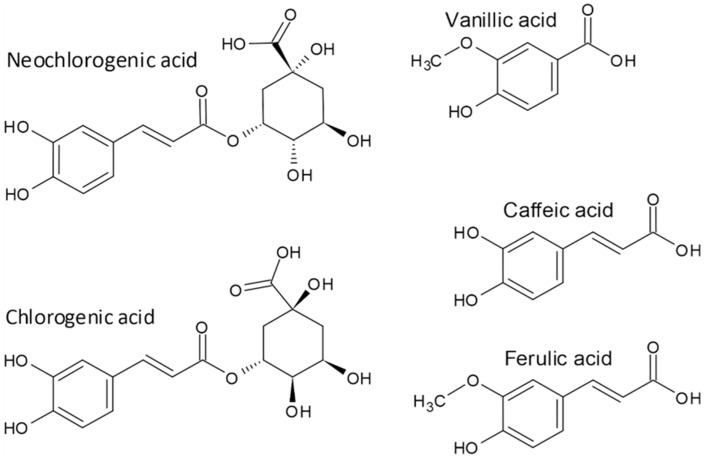
Buckwheat phenolic acids.

Native and germinated buckwheat is a source of phenolic acids such as neochlorogenic acid, chlorogenic acid, vanillic acid, caffeic acid, and ferulic acid (Figure 4) [87,88]. Compared to raw Tartary buckwheat, the contents of phenolic acids in fermented Tartary buckwheat are increased. Podolska et al. [89] reported that total phenolic acid contents are higher in Tartary buckwheat than in common buckwheat. The concentration of neochlorogenic acid was higher in the non-treated Tartary buckwheat grain than in the hydrothermally treated flour-water mixtures [90], meaning that neochlorogenic acid is degraded during the dough making. However, neochlorogenic acid concentrations were maintained if the temperatures of hydrothermally treated samples were at least 80 °C. So, the Tartary buckwheat grain enzymes are in the mixture of flour and water at the moderate temperature transforming neochlorogenic acid, but the initial treatments with high-temperature enzymes are inactivated [90]. The transformation of other phenolic acids during the treatment of Tartary buckwheat flour or grain is, according to the knowledge of present authors, not yet investigated.

Noratto et al. [91] reported that neochlorogenic and chlorogenic acids have potential as chemopreventive dietary compounds because they have shown relatively high growth inhibition on an estrogen-independent breast cancer cell line. Neochlorogenic acid might be a colon cancer suppressive plant component [91,92].

## 4. Tartary Buckwheat Flour Products

The typical buckwheat flour product is bread, prepared in the central part of Europe at least since 1689 [93]. It is not known if common or Tartary buckwheat, or a mixture of both, was used for bread. It is not known when Tartary buckwheat was introduced to central Europe, but it is documented that it was widely cultivated several years after 1816, when weather conditions were not favorable for other crops [14]. In Tartary buckwheat bread making experiments from an initial 7 mg of rutin per g DM in buckwheat flour, bread contained 2 mg of rutin per g DM. Additionally, 6 mg of quercetin appeared in the bread as a result of the enzymatic decomposition of rutin [19,20]. Rutin is better conserved in Tartary buckwheat flour products if the Japanese trace-rutinosidase variety of Tartary buckwheat ‘Manten-Kirari’ is used for making Tartary buckwheat flour food products [20,23].

Tartary buckwheat was used in experimental bread made from 10% chia seeds and 90% Tartary buckwheat (with total flavonoids 22.2 mg rutin equivalents per g flour, dry matter). In the resulting bread, there was 16.1 mg of total flavonoids, expressed in rutin equivalents per g of bread, or 16.8 rutin equivalents per gram of bread without the addition of chia [94].

Dark and light buckwheat flour differ in composition [8,95]. Dark buckwheat flour and bran contain more proteins, fiber, secondary metabolites (e.g., rutin), and more mineral substances compared to light buckwheat flour [96,97]. Very bright buckwheat flour contains mostly intact starch grains and has water-repellent properties. Unlike large wheat starch grains, buckwheat starch grains are not easily damaged during grinding [98]. The use of light buckwheat flour for mixtures of buckwheat and wheat flour does not improve the taste of bread, while dark buckwheat flour improves its taste [99].

Buckwheat bread is normally made with approximately 30% of buckwheat flour, and the rest is wheat flour. One possibility is to make buckwheat bread dough and add Tartary buckwheat groats (kasha). In such a case, it is best to use buckwheat kasha obtained by traditional technology to husk pre-cooked buckwheat grain.

At the Education Centre, Piramida Maribor, they developed several food products from Tartary buckwheat flour, namely various dumplings, gluten-free Tartary buckwheat bread, Tartary buckwheat sticks, diverse Tartary buckwheat potica, Tartary buckwheat pasta, and other products. Some of these recipes are described in the bilingual (Slovenian-English) book *Ajda-Buckwheat* [95].

Buckwheat phenolic compounds can inhibit fungal development due to the phenolic hydrophobic interactions with cell membranes [100]. This effect is important for the antifungal properties of sourdoughs. Lactic acid bacteria can split flavonoid glycosides to flavonoid aglycones and sugar and can further metabolize aglycones. The resulting metabolites, which include lactic acid and other organic acids, also serve to increase the antifungal activity of buckwheat sourdough. This might explain the prolonged shelf life of Tartary buckwheat sourdough bakery products.

In Asia, and in some places in Europe (Slovenia, Italy, France) noodles are popular traditional buckwheat food products, either freshly made or as dry industrial products [14]. However, in Japan, Korea, and China, Tartary buckwheat noodles are produced in some places as well [12,101]. Cooking Tartary buckwheat noodles at a temperature lower than 80 °C is an adequate way to save the flavonoids in Tartary buckwheat pasta from loss [101].

Approximately 90% of rutin in pasta from ‘Manten-Kirari’ Tartary buckwheat cultivar remained unchanged. ‘Manten-Kirari’ noodles exhibited only slight bitterness while the control variety showed the strong bitterness of quercetin, the degradation product of rutin. The new Tartary buckwheat variety ‘Manten-Kirari’ is promising for making Japanese buckwheat (soba) rutin-rich noodles with minimal bitterness [102].

## 5. Tartary Buckwheat Groats

Preparing husked buckwheat grain, kasha, is very challenging. The process has been known for a long time, as it was described by Valvasor in 1689 [93]. To husk buckwheat grain, first they must be soaked in boiling water; during this treatment the starch swells. When ready, the water is removed, and the grain is dried at a moderate temperature. It should be dried only so that the husk is dry and brittle, and the inside of the grain is elastic but solid enough not to squeeze or smear when the process is continued. Properly dried grain is placed into a husking device.

The modern husking of buckwheat grain is essentially the same as it used to be—cooking, drying, husking, again drying, and blowing off the husk. Buckwheat kasha is obtained the traditional way by pre-cooking it before husking so it has a special taste and properties [14].

Another method to husk buckwheat is to use non-precooked grain. The raw husked common buckwheat groats are green while the just-harvested buckwheat was husked. After some weeks, the chlorophyll fades and phenolic substances oxidize, so the older non-precooked buckwheat groats are yellow, and later become reddish. By the green color, it is possible to estimate that the raw husked groats are fresh and made from just-harvested common buckwheat [14]. Buckwheat groats obtained by pre-cooking are harder, and by pressure break into larger particles with a vitreous appearance. Pre-cooked buckwheat groats are cooked nicely, and they also have a special taste. The substances of traditional buckwheat groats, important for this distinctive taste, were studied [103]. Buckwheat groats are popular in Slovenia, Croatia, Poland, Belarus, Ukraine, Russia, and in some parts of China. Many dishes can be made with common or Tartary buckwheat kasha [95].

Tartary buckwheat has thick husks and it is difficult to remove them when obtaining groats. However, rice-Tartary buckwheat is a form of Tartary buckwheat with less firmly attached husks. It is used in breeding programs to obtain higher levels of flavonoids, including rutin and quercetin [44].

When buckwheat groats are repeatedly heated and cooled down, the starch molecules change their structure; they are retrograded and become not easily digestible by our enzymes [104,105,106]. Thus, retrograded starch is a part of dietary fiber. There are microflora in the colon, which have enzymes to break down retrograded starch molecules. The starch is broken down here into short fatty acids, which have a beneficial effect on the cells of the colon [106]. Eating buckwheat kasha also improves insulin response and prolongs satiety sensation [106,107]. In buckwheat dishes, especially in Tartary buckwheat dishes, are also metabolites, such as tannins and quercetin, that inhibit starch degradation during digestion [108,109].

## 6. Green Parts of Tartary Buckwheat Plants

**Figure 5:**
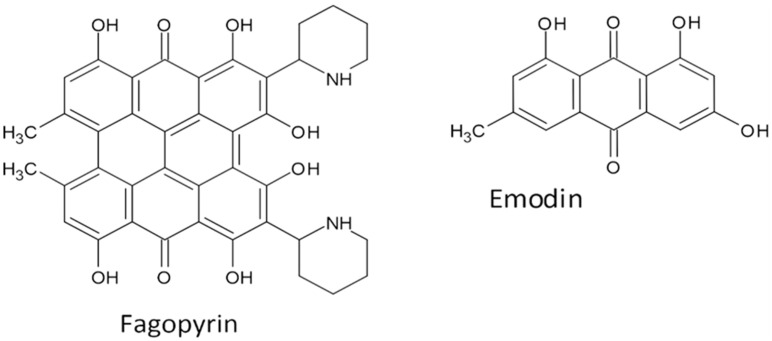
Fagopyrin and emodin.

In the green parts of Tartary buckwheat plants is an anthraquinone secondary metabolite fagopyrin (Figure 5). Among buckwheat’s secondary metabolites, fagopyrin appears to present a potential health threat when the green parts of the plants are consumed. The ingestion of buckwheat grain and resulting grain food products has been shown to be safe due to the low concentrations of fagopyrin (Table 3) [11,110,111,112,113,114]. It is reported that it is possible to use fagopyrin as a protective substance against Phytophthora [115]. The other anthraquinone metabolite in Tartary buckwheat is emodin [116] (Figure 5). Tartary buckwheat emodin is concentrated in leaves and grain, but less in the stem and cannot be detected in roots [117,118]. Emodin has some antiviral effects and was identified that it may be used for clinical trials [119].

**Figure 6:**
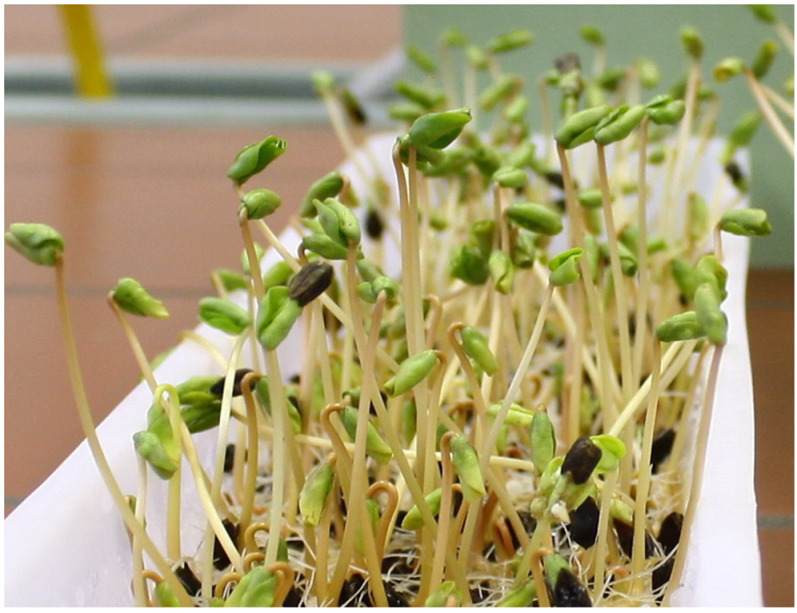
Tartary buckwheat sprouts (Photo Dr. Paula Pongrac).

Tartary and common buckwheat sprouts (Figure 6) are popular in Korea, mainly as an addition to buckwheat noodle dishes or as a filling for buckwheat pancakes [120,121]. Tartary buckwheat sprouts have higher biological activity and are a good dietary source of phenolic and flavonoid compounds, especially for rutin and antioxidant activity compared to common buckwheat sprouts [122,123,124,125,126].

The rutin concentration in Tartary buckwheat sprouts does not depend on the mineral element composition of water in sprouts, but the concentration of quercetin and catechin does. While Tartary buckwheat grain contained 914 mg rutin per kg dry weight, resulting sprouts contained approximately 10,000 rutin per kg dry weight. In grain and sprouts, it was approximately 1270 of quercetin in mg per kg, and in sprouts approximately 700–1100 mg per kg, depending on the mineral element composition of the water. In grain and sprouts, it was approximately 4510 mg per kg of catechin, and in sprouts approximately 7000–8000 mg per kg, again depending on the mineral element composition of the water [127,128].

Adequate light and sucrose and calcium chloride treatment have a significant effect on the growth of buckwheat sprouts. Shin et al. and Nam et al. [129,130] investigated the influence of different light qualities on the synthesis of phenolic compounds, antioxidant activity, rutin content, free amino acids, and vitamin C in both types of buckwheat sprouts. Sucrose and calcium chloride treatment in buckwheat sprouts resulted in the significant accumulation of bioactive compounds such as polyphenols, flavonoids, vitamins C and E, and antioxidant activity, without negatively affecting sprout growth [131]. The number of individual metabolites is also greatly influenced by the age of the sprouts. Yang et al. [132] showed a chemical difference between the 3-day-old and 8-day-old sprouts using orthogonal partial least squares analysis (OPLS–DA). Twenty-seven metabolites were higher in the 3-day-old sprouts and only three metabolites were higher in the 8-day-old sprouts. A total of 25 differential compounds were all significantly upregulated upon UV-B radiation, especially for epicatechin.

## 7. Conclusions

Tartary buckwheat metabolites are analyzed using diverse combinations of methanol, ethanol, and water extraction. The most convenient and widely used are HPLC methods, but capillary electrophoresis can be used as well.

For the identification of metabolites in diverse Tartary buckwheat tissues, it is possible to develop the potential application of micro-proton induced X-ray emission (micro-PIXE), synchrotron-based micro-X-ray fluorescence (micro-XRF), inductively coupled plasma-mass spectrometry (ICP-MS), and other recently developed physical methods. In this way it is possible to reveal a connection of plant structures, their phytochemical constitution, and bioactivities. The information on the phytochemical composition of the details of Tartary buckwheat plant structures by bioimaging and other newly developed methods can provide the basis for obtaining the optimal nutritional and functional value of final products for grain milling and processing. A recently developed electrochemical sensor for rutin determination will be of much help in the simple determination of rutin in Tartary buckwheat products. Another challenge is to develop electrochemical sensors for other Tartary buckwheat bioactive substances.

Studies of the potential bioactivities of Tartary buckwheat metabolites include computer simulation methods by molecular docking performed in silico, in vitro methods on human tissues, experiments with laboratory animals, and epidemiological and clinical studies.

The capability of Tartary buckwheat to tolerate high altitudes and high levels of UV radiation is due to several groups of genes involved in signal transfer and gene regulation for the synthesis of secondary metabolites.

Flavonoids are compounds of special interest in food products due to their antioxidant properties and potential in preventing tiredness, diabetes mellitus, oxidative stress, and neurodegenerative disorders such as Parkinson’s disease. Important bioactivities of quercetin are connected with the ability of quercetin to cross the blood-brain barrier and accumulate in the brain. Some Tartary buckwheat metabolites are transformed during the processing and preparation of food products. In plants and food products, the appearance of quercetin is mainly the result of the enzymatic degradation of rutin due to rutinosidase activity.

Buckwheat groats (kasha) are, during the production and preparation of food, repeatedly heated and cooled down; the starch molecules change their structure and become retrograded and slowly digestible by human enzymes. Retrograded starch is a part of dietary fiber. There are microflora in the colon, which produce enzymes to break down retrograded starch molecules. Starch is broken down in the colon into short fatty acids, which have a beneficial effect on the colon cells. Eating buckwheat kasha also improves insulin response and prolongs satiety sensation. In buckwheat dishes, especially in Tartary buckwheat dishes, are also metabolites, such as tannins and quercetin, that inhibit starch degradation during digestion.

## Figures and Tables

**Table 1:** Content of total polyphenols and flavonoids isolated from different parts of Tartary buckwheat (/: no data, BTB: black Tartary buckwheat accessions, YTB: yellow Tartary buckwheat accessions).

Compounds Name	Content in Dry Weight					References
	Roots	Stem	Leaves	Flowers	Seeds	
Total polyphenols (mg/g)	19.76	/	32.51	/	/	[49]
Total polyphenols (µg/mg)	/	96.5–109.8	/	/	208	[50]
Total flavonoids (mg/g)	/	17	100	160	/	[51]
Total flavonoids (%)	/	/	/	/	2.04	[52]
Total flavonoids (µg/mg)	/	38.1	/	/	142.2	[50]
Total flavonoids (mg/g)	/	/	76.40	145.4	20.24	[53]
Total flavonoids YTB (mg/g)	/	/	/	49.07	/	[54]
Total flavonoids BTB (mg/g)	/	/	/	52.81	/	[54]
Total flavonoids (mg/g)	/	/	213.66	/	/	[55]

**Table 2:** Content of flavonols isolated from different parts of Tartary buckwheat (/: no data, BTB: black Tartary buckwheat accessions, YTB: yellow Tartary buckwheat accessions).

Compounds Name	Content in Dry Weight					References
	Roots	Stem	Leaves	Flowers	Seeds	
Rutin (mg/g)	/	/	/	/	16.7	[73]
Rutin (%)	/	/	/	/	0.8–1.7	[5]
Rutin (g/100 g)	/	/	/	/	1.83–1.97	[74]
Rutin (g/100 g)	22.3	482.6	2876.0	3518.6	1469.8	[75]
Rutin (mg/g)	/	/	/	/	8.68–13.34	[76]
Rutin (mg/g)	/	/	/	/	7.56–8.9	[77]
Rutin (mg/g)	/	/	/	/	11.99–21.4	[66,78]
Rutin (%) different varieties	/	/	/	/	1.19–2.91	[79]
Rutin (mg/g)	/	/	/	/	16.69	[52]
Rutin (mg/g) different varieties	/	/	/	/	6.5–16.64	[72]
Rutin (mg/g)	/	/	/	/	18.08–18.53	[80]
Rutin (mg/g)	/	/	/	/	14.1	[81]
Rutin (mg/g)	/	/	/	/	11.97	[50]
Rutin (%)	/	/	6.06 %	7.77 %	1.35 %	[53]
Rutin (mg/g)	/	/	/	/	14.1	[82]
Rutin (mg/g)	0.8	3.0	28	38	18	[42]
Rutin (mg/mg) YTB	/	/	/	35.93	/	[54]
Rutin (mg/mg) BTB	/	/	/	38.80	/	[54]
Rutin (μg/g)	1963.4		2949.3	2253.8	/	[83]
Rutin (mg/g)	3–8	6–14	/	47–63	/	[46]
Quercetin (mg/g) different varieties	/	/	/	/	0.47–0.9	[72]
Quercetin (μg/g)	7.2	2.1	172.1	844.7	/	[83]
Quercetin(mg/mg) YTB	/	/	/	3.06	/	[54]
Quercetin(mg/mg) BTB	/	/	/	6.49	/	[54]
Kaempferol(mg/mg) YTB	/	/	/	0.09	/	[54]
Kaempferol(mg/mg) BTB	/	/	/	0.06	/	[54]
Myricetin (mg/mg) YTB	/	/	/	0.40	/	[54]
Myricetin (mg/mg) BTB	/	/	/	0.43		[54]
Catechin (mg/mg)	/	/	/	/	0.12	[84]
Catechin (μg/g)	5.3	1.1	9.9	11.9	/	[83]
Epicatechin (mg/g)	/	/	/	/	0.04	[84]

**Table 3:** Content of vitexin and fagopyrins isolated from different parts of Tartary buckwheat (/: no data).

Compounds Name	Content In Dry Weight					References
	Roots	Stem	Leaves	Flowers	Seeds	
Vitexin (μg/g)	5.0	/	3.3	42.2	/	[83]
Fagopyrins (μg/g)	/	/	/	38.2	/	[83]
Fagopyrins (mg/g)	/	2	0.56	6.08	/	[112]

## Data Availability

The data presented in this study are available on request from the first and corresponding author.
